# *QuickStats:* Age-Adjusted Percentage* of Adults Aged ≥18 Years Who Were Ever Tested for Human Immunodeficiency Virus (HIV)^†^ Infection, by U.S. Census Region^§^ — National Health Interview Survey, 2017**^¶^**

**DOI:** 10.15585/mmwr.mm675152a6

**Published:** 2019-01-04

**Figure:**
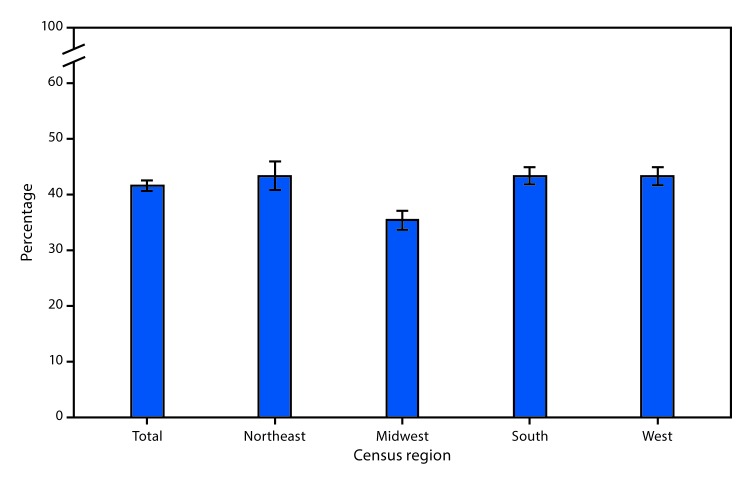
In 2017, 41.7% of adults aged ≥18 years had ever been tested for HIV. Adults living in the Midwest (35.5%) were less likely to have ever been tested for HIV than adults in the Northeast (43.5%), South (43.5%), and West (43.4%).

**Source:** Tables of Summary Health Statistics, 2017. https://ftp.cdc.gov/pub/Health_Statistics/NCHS/NHIS/SHS/2017_SHS_Table_A-20.pdf.

